# Sacubitril/Valsartan in the Treatment of Heart Failure With Reduced Ejection Fraction Focusing on the Impact on the Quality of Life: A Systematic Review and Meta-Analysis of Randomized Clinical Trials

**DOI:** 10.7759/cureus.48674

**Published:** 2023-11-11

**Authors:** Priyadarshini Bhattacharjee, Zahid Khan

**Affiliations:** 1 Medicine, University of Cambridge, Cambridge, GBR; 2 Acute Medicine, Mid and South Essex NHS Foundation Trust, Southend-on-Sea, GBR; 3 Cardiology, Barts Heart Centre, London, GBR; 4 Cardiology and General Medicine, Barking, Havering and Redbridge University Hospitals NHS Trust, London, GBR; 5 Cardiology, Royal Free Hospital, London, GBR

**Keywords:** six-minute walking distance or exercise, sacubitril/valsartan, heart-related quality of life (hrqol), nyha classes ii and iii, left ventricular systolic dysfunction, systematic review on heart failure with reduced ejection fraction, exercise tolerance and heart failure, heart failure and quality of life, entresto, heart failure with reduced ejection fraction

## Abstract

There exists a paucity of research data reported by analyses performed on randomized clinical trials (RCTs) that encompass quality of life (QOL) and the aftermath for patients suffering from heart failure with reduced ejection fraction (HFrEF). This systematic review and meta-analysis of randomized clinical trials (RCTs) have been done to evaluate the drug sacubitril/valsartan in the treatment of heart failure (HF) with reduced ejection fraction (HFrEF) with a clear focus on the effect it bestows on measures of physical exercise tolerance and quality of life. A thorough systematic search was done in databases including Cochrane Central Register of Controlled Trials (CENTRAL), ClinicalTrials.gov, Embase, and PubMed from 1 January 2010 to 1 January 2023. The search only included published RCTs on adult patients aged 18 and above, with heart failure with reduced ejection fraction (HFrEF). Data analysis was performed by using the software RevMan 5.4 (Cochrane Collaboration, London, United Kingdom). The included studies' bias risk was assessed using the Cochrane Collaboration's Risk of Bias tool. The quality of evidence for the primary outcome was done using the Grading of Recommendations, Assessment, Development, and Evaluations (GRADE) framework.

This systematic review and meta-analysis of RCTs yielded 458 studies, of which eight randomized clinical trials were included and analyzed. The meta-analysis of the included trials shows that the I^2^ value is 61% (i.e., I^2^ > 50%), demonstrating a substantial heterogeneity within the studies. The left ventricular ejection fraction (LVEF) expressed in percentage was reported in the five studies, and thereby, a subgroup analysis that yielded a confidence interval (CI) of 95% had the standard mean difference of 0.02 (-0.02, 0.07). The trials had disparity between the reporting of effect on peak oxygen consumption (VO_2_), measured through cardiopulmonary exercise testing (CPET) methods, six-minute walking test (6MWT), overall physical activity, and exercise capacity. Sacubitril/valsartan did not exponentially improve peak VO_2_ or 6MWT in these trials; however, the patient-reported data suggested that the quality of life was modestly influenced by the drug. A subgroup analysis was performed using the pooled effect value by the random effects model. The findings showed that the sacubitril/valsartan group significantly was better than the control group in improving HFrEF-associated health-related quality of life (HRQoL).

This study is a systematic review and meta-analysis of randomized clinical trials that evaluated the drug sacubitril/valsartan in treating heart failure with reduced ejection fraction (HFrEF) and focused on its tangible effect on the measures of physical exercise tolerance and quality of life. It depicts that the statistical scrutiny due to the lack of significant data and parity across studies did not impart significant improvement of either LVEF, peak VO_2_, or 6MWT with the use of sacubitril/valsartan; however, the reported exercise tolerance, including daytime physical activity, had a modest impact with the said drug. The pooled values demonstrated that the sacubitril/valsartan group significantly outperformed the control group in improving HFrEF HRQoL.

## Introduction and background

An operational or anatomical cardiac problem that impairs ventricular refilling or blood ejection to the systemic circulation causes heart failure (HF), a complicated medical condition. In essence, it is an inability to satisfy the underlying needs of circulation. Internationally, heart failure continues to be a condition with a significant death and disability rate [1]. It is predicted to affect 26 million individuals worldwide and has an impact on healthcare costs.

Heart failure is classified according to its manifestations and determined left ventricular ejection fraction (LVEF). Heart failure with reduced ejection fraction (HFrEF), heart failure with preserved ejection fraction (HFpEF), and heart failure with midrange ejection fraction (HFmrEF) are the three subtypes of heart failure caused by left ventricular dysfunction. The latter could include a variety of left ventricular dysfunction (a congruent effect of systolic and diastolic heart failure) [2].

Etiology of heart failure

Heart anatomical anomalies, cardiac functioning aberrations, and other initiating variables can contribute to congestive heart failure (CHF). In the past, myocardial infarction and coronary heart disease accounted for a vast majority of instances. The two strongest predictors for cardiovascular disease today are coronary artery disease and diabetes mellitus. Hypertension, valvular heart disease, uncontrolled arrhythmia, myocarditis, and congenital heart disease are other intrinsic causes of congestive heart failure (CHF). In addition to the obvious etiologies, restrictive cardiomyopathies and constrictive pericarditis can result in diastolic heart failure with poor ventricular filling [3].

It is indeed critical to understand the causes of decompensated heart failure because they are mostly responsible for the incidence and death brought on by the condition. Reduced regular exercise, sodium limitation in the food, and incorrect medication therapy are the three main causes of decompensated congestive heart failure. The second most prevalent factor leading to decompensated heart failure is persistent hypertension [4]. In individuals with underlying congestive heart failure, unregulated tachyarrhythmias can quickly cause the worsening of the condition.

Elevated heart failure is characterized by a different spectrum of disorders connected to "congestive heart failure." By interpretation, this is not a myocardial function defect but rather the heart's inability to cope with the heightened systemic demands brought on by extracardiac illnesses. Severe anemia, thyrotoxicosis, obesity, nutritional deficiencies (such as thiamine deficiency), and pregnancy are common causes of this form of congestive heart failure [5].

Epidemiology of heart failure

Heart failure (HF) is a widespread healthcare concern that impacts roughly 2% of Westerners, with frequency rising significantly from 1% in those under the age of 40 to 10% in adults over the age of 75 [6].

From 2013 to 2016, there were 6.2 million clinically evident cases of heart failure in the United States [7]. According to some statistics, the incidence rate has hit a ceiling; nonetheless, as more people have received treatment, the frequency rises. It has not resulted in people with heart failure having a higher quality of life (QOL) or experiencing fewer admissions. The Global Health Data Exchange registry estimates that there are currently 64.34 million cases of CHF worldwide. This amounts to 346.17 billion US dollars spent on healthcare and 9.91 million years lost due to disability (YLDs) [8]. Ischemia-induced cardiomyopathy is a significant contributor to HF in developing nations, coupled with high blood pressure [9].

Supported by small research studies from such countries, the most noticeable variation is a higher prevalence of isolated right heart failure. Theoretically, this is caused by the increased frequency of pulmonary, heart, and tuberculous diseases. Strong evidence is lacking to support such statements [1]. The scientific information and consequences in people with symptomatic HF from Europe and the United States have been reported by the EuroHeart Failure Survey, the Acute Decompensated Heart Failure National Registry (ADHERE), and the Organized Program to Initiate Lifesaving Treatment in Hospitalized Patients With Heart Failure (OPTIMIZE-HF) [10-13].

Pathophysiology of heart failure

Despite attempting to sustain acceptable cardiac efficiency, the adaptive mechanisms that may be sufficient to keep the heart's overall contractile function at a rough baseline state become problematic.

Some compensatory mechanisms are used by cardiac physiology to modify during the early stages of congestive heart failure to sustain blood flow and satisfy systemic mandates. This encompasses cardiac hypertrophy, the Frank-Starling mechanics, modifications in myocyte renewal, and myocardial hypercontractility. The myocardium tries to make up for the incremental wall stress through eccentric remodeling, but this only worsens the loading circumstances and wall stress [14].

The neuroendocrine system is stimulated by a decline in blood pressure, which results in the production of epinephrine, norepinephrine, endothelin-1 (ET-1), and vasopressin. Vascular constriction brought on by them increases afterload. Cyclic adenosine monophosphate (cAMP) levels rise, which in turn leads the myocytes' cytosolic calcium levels to rise. As a result, myocardial relaxation is further inhibited, and myocardial contractility is increased [14].

Myocardial oxygen uptake rises as a result of an increment in afterload, cardiac contractility, and poorer myocardial relaxation. Myocardial cell loss and apoptosis ultimately occur from this contradictory requirement for higher cardiac output to meet myocardial needs. An ongoing loop of heightened neurohumoral excitement, decreased cardiac output, and unfavorable hemodynamic and myocardial responses results from the progression of apoptosis [14].

Together with increasing salt and water accumulation and vasoconstriction, a decrease in cardiac output also stimulates the renin-angiotensin-aldosterone system (RAAS). This intensifies the heart's dysfunctional processes, which lead to progressive heart failure. The RAAS also secretes angiotensin II, which has been linked to an increase in interstitial fibrosis and cardiac cellular hypertrophy. This one has demonstrated that angiotensin II's detrimental effect causes myocardial remodeling to rise [15].

History and physical criteria of heart failure

Heart failure is typically diagnosed and categorized based on the presence/severity of symptoms and the results of a physical examination. To properly treat the patient, it is critical to gather a thorough history of symptoms, underlying medical issues, and functional capacity/exercise endurance. Breathlessness is the symptom that is most frequently mentioned [1].

To clarify the potential causes of heart failure and choose the patient's treatment strategy, this complaint must be further qualified. It is necessary to categorize shortness of breath even further to ascertain whether it is transient or persistent and related to effort or postural changes (orthopnea) or both. Other manifestations of HF that are frequently mentioned include weariness, anorexia, tachycardia, and chest pain. Recumbent coughing, which some patients may present with, may be caused by orthopnea [1].

Patients with heart failure require a thorough evaluation during the physical examination. Anxiety, diaphoresis, and poor nutritional status are among the general symptoms of individuals with severe, chronic heart failure or those with abruptly decompensated heart failure. The traditional diagnosis of pulmonary rales corresponds to moderate to severe heart failure [1].

In cases of acute decompensated heart failure, wheezing may be evident. A mucus that is foamy and has a bloody tint may be seen as lung congestion becomes more severe. It is crucial to understand that respiratory congestion is not automatically ruled out by the lack of rales. A further common feature that all HF individuals must have evaluated is jugular venous distention [16]. The Kussmaul sign, a paradoxical rise in jugular venous distention with breathing, can be noted. Hepatojugular reflux (the distention of the jugular vein following the application of pressure on the liver with the patient lying at a 45° angle) will be evident in patients with elevated left-sided filling pressures. When there is a significant amount of volume overload, peripheral edema, which is evident in severe heart failure, could be seen [16]. Third heart sound (S3) gallop, pulsus alternans, and the accentuation of P2 are cardiac symptoms in HF sufferers. The most important and earlier discovery related to HF is an S3 gallop [17]. The presence of mitral and tricuspid regurgitation murmurs is a sign of decompensated dilated cardiomyopathy [1].

Diagnostic criteria for heart failure

According to the widely accepted Framingham Diagnostic Criteria for Heart Failure, the condition must meet either two main criteria or one major and two minor eligibility requirements to be diagnosed as having heart failure. Although this testing method has very low specificity, it is extremely sensitive for the diagnosis of heart failure [18].

The major criteria include acute pulmonary edema, cardiomegaly, hepatojugular reflex, neck vein distention, paroxysmal nocturnal dyspnea or orthopnea, pulmonary rales, third heart sound (S3) gallop, weight loss of 4.5 kg or more in five days in response to treatment, central venous pressure greater than 16 cm of water, and radiographic cardiomegaly.

The minor criteria include ankle edema, dyspnea on exertion, hepatomegaly, nocturnal cough, pleural effusion, tachycardia (heart rate greater than 120 beats per minute), and a decrease in vital capacity by one-third of the maximal value recorded.

The New York Heart Association (NYHA) system does a poor performance of differentiating between HF patients with various levels of perceived disability. Studies done on trials discovered that the difference between NYHA classes II and III is a poor predictor of negative consequences in heart failure and fails to distinguish between patients across the breadth of functional disability and recognized that biomarkers such as N-terminal pro-B-type natriuretic peptide (NT-proBNP), the Kansas City Cardiomyopathy Questionnaire (KCCQ), and the Minnesota Living with Heart Failure Questionnaire (MLHFQ) and other markers of pathogenicity and patient-reported experiences may be more suitable for guiding recruitment methods and evaluating the effects of treatment approaches on clinical conditions [19]. The classes are detailed as class I, symptom onset with more than ordinary level of activity; class II, symptom onset with an ordinary level of activity; class III, symptom onset with minimal activity; class IIIa, no dyspnea at rest; class IIIb, recent onset of dyspnea at rest; and class IV, symptoms at rest.

Evaluation of heart failure

To determine the source and/or degree of heart failure, a thorough scientific investigation is required, including testing for anemia, iron deficiency, renal dysfunction, and liver dysfunction. In individuals with persistent HF, serum osmolarity has a predictive value as a predictor of mortality [20]. They assist in predicting short-term mortality for patients with decompensated heart failure who are hospitalized. Sufferers of HF who presented with hyponatremia had a considerably higher chance of not only dying in the hospital but also dying within 30 days, according to the findings of the OPTIME-CHF trial. Individuals who participated in the study had a serum concentration of sodium level of 134 mEq/L. Those with the lowest percentile of serum sodium levels at admission had the greatest chance of death [20].

In individuals with equivocal manifestations, serum levels of B-type natriuretic peptide (BNP) or N-terminal proBNP (NT-proBNP) can help distinguish between cardiac and noncardiac reasons for dyspnea. BNP is utilized to determine the rates of dying in individuals with heart failure since it is a significant determinant of elevated left ventricular end-diastolic pressure. BNP levels are used principally as a measure to evaluate treatment effectiveness and are correlated with New York Heart Association (NYHA) categorization [21,22]. Natriuretic peptides should not be the main focus of a patient's therapy regimen if they have a true clinical presentation of heart failure. It is critical to keep in mind that elderly individuals, persons with atrial fibrillation, and those with kidney disease can all have increased levels of BNP and NT-proBNP. On the other hand, cases of severe heart failure, hypothyroidism, and obesity may have deceptively low BNP levels precisely due to myocardial fibrosis [21,22].

Transthoracic echocardiography (TTE) can identify the concentration of localized wall motion abnormalities or valvular pathology, evaluate for systolic and diastolic malfunction, and provide light on these conditions. However, it can be challenging to provide enough acoustic windows for people who have severe obesity, are pregnant, or use mechanical ventilation. These patients may benefit from other tests such as cardiac magnetic resonance imaging (MRI) or transesophageal echocardiography. The gold standard criterion for determining right ventricular (RV) function is cardiac MRI [23].

Another method, despite the price, for assessing the functioning of the left and right ventricles is the radionuclide multiple-gated acquisition (MUGA) scan. When there has been a discrepancy in the ejection fraction (EF) readings from several investigations, patients will have a MUGA scan, which is the most reliable inspection to quantify ejection fraction (EF) [24]. An additional therapeutic method for evaluating EF, regional wall motion, and regional wall thickening is myocardial perfusion imaging, which is electrocardiogram (ECG)-gated [24].

Scintigraph imaging with iobenguane I 123 injectable is known as iobenguane scanning. It has already been administered to individuals with an LVEF of around 35% and New York Heart Association (NYHA) classes II-III to determine their cardiac risk. Another homolog of norepinephrine is iobenguane I 123. The said examination can reveal how much norepinephrine is being taken up by the heart's sympathetic nerves. A better forecast is connected to norepinephrine reuptake that is strengthened [25]. Cardiovascular catheterization, stress testing, and electrocardiograms are further examinations carried out on HF patients. To ascertain the underlying cause of the illness, they are utilized in HF patients. They are not specifically involved in the diagnosis or prognosis of HF. Electrocardiogram (ECG) anomalies in patients with severe systolic CHF are given. Nonetheless, the ECG may be normal in HFpEF sufferers [1].

Heart failure with reduced ejection fraction

Heart failure with reduced ejection fraction (HFrEF) is a form of heart failure that is characterized by a lower left ventricular ejection fraction and is one of the three types of heart failure [26,27]. It is caused by many different diseases, including ischemia, pressure overload, volume overload, cytotoxic medications, and arrhythmias [28], and happens to be associated with a reduction in stroke volume and cardiac output [26], and it is defined by an ejection fraction (EF) below 40% [28]. Heart failure with reduced ejection fraction (HFrEF) is diagnosed based on the left ventricular ejection fraction (LVEF). Generally, EF is measured by echocardiography and is defined as a value between 0% and 100%. HFrEF is diagnosed when the EF is less than or equal to 40%, while heart failure with midrange ejection fraction (HFmrEF) is diagnosed when EF is between 40% and 49% or 41% and 49% [29].

Studies have shown that changes in left ventricular ejection fraction over time are associated with the risk of all-cause mortality or hospitalization for heart failure [27]. HFrEF is also associated with a higher prevalence of ischemic heart disease and frequent renal impairment and is a major parameter for the diagnosis, phenotyping, prognosis, and treatment decisions of heart failure [27]. The current European Society of Cardiology (ESC) recommendations provide clear guidelines on how to treat HFrEF, and the treatment for this condition is based on certain substance classes [26].

Angiotensin-converting enzyme inhibitors (ACEIs), angiotensin receptor blockers (ARBs), beta-blockers, aldosterone antagonists, and device treatments have all been used in numerous major randomized controlled trials over the past few decades to show better survival in HF patients [30-32]. Therapy with mineralocorticoid receptor antagonist (MRA), angiotensin receptor-neprilysin inhibitor (ARNI), or sodium-glucose cotransporter-2 inhibitor (SGLT2i) may have potential benefits in patients with HFrEF according to post hoc and subgroup analyses of heart failure trials [27]. Elevated heart rate in heart failure patients is associated with poor ventricular function and is repeatedly associated with a poorer prognosis [26]. This occurs since reduced ejection fraction in heart failure leads to an increase in heart rate, which in turn causes reduced cardiac output [26]. Non-cardiovascular mortality is lower in patients with HFrEF compared to patients with HFpEF; however, cardiovascular mortality is higher in patients with HFrEF compared to patients with HFmrEF and HFpEF [27].

Heart failure (HF) is a clinical illness that worsens with time and significantly lowers the quality of life (QOL). It is crucial to assist patients in maintaining their ideal QOL. Although definitions in the literature vary greatly and few consider the participant's perspective, QOL represents individuals' genuine impressions regarding the influence of a clinical experience of illness on ordinary living [33].

Epidemiological data suggest that there is a huge burden of HF in the world [34]. Survivors' ability to exercise and their health-related quality of life (HRQoL), which has a big impact on daily life, are thought to be affected by a load of complaints and the incapacitating effects of HF. Affected individuals have much lower life quality than patients with other chronic diseases, which warrants greater clinical care [35,36].

Over the previous two decades, advances have been made in both the therapeutic and diagnostic aspects of HF treatment. Individuals with chronic HF and a low ejection fraction experienced a reduction in mortality rate when taking an angiotensin receptor-neprilysin inhibitor. The imbalance between the renin-angiotensin-aldosterone and natriuretic peptide systems is reduced by sacubitril/valsartan (LCZ696). The clinical effectiveness, practical experience, tolerability, validity of the etiology of cardiomyopathy, gender differences, and regulatory affairs of LCZ696 in the treatment of patients with HF with reduced ejection fraction have all been examined in previous studies [37]. Sacubitril/valsartan has since grown in importance as an area of study in the treatment of heart disease as time goes on, and it may one day be crucial in the overall system integration of the cardiac incident cascade.

## Review

Methodology

Less attention has been paid to the overall aspect of health-related quality of life, which happens to be one of the main goals of HF treatment. In addition, earlier investigations provided different outcomes. It is currently unknown how sacubitril/valsartan affects the HRQoL. This study set out to undertake a thorough quantitative analysis of the data from recently published randomized clinical trials (RCTs) to determine how sacubitril/valsartan affected the quality of life in patients with HFrEF, which has previously been less explored. The methodology of this systematic review and meta-analysis is discussed in detail below.

The Search Strategy Used in the Study

A systematic search was made in the databases including PubMed, Embase, ClinicalTrials.gov, and Cochrane Central Register of Controlled Trials (CENTRAL) to look for clinical trials relevant to the topic of interest. The search was a combination of Medical Subject Headings and keywords in the English language. The terms included were "heart failure," "HF," "heart decompensation," "heart insufficiency," "heart incompetence," "entresto," "sacubitril/valsartan," "valsartan/sacubitril," "sacubitril plus valsartan," "valsartan plus sacubitril," "sacubitril and valsartan sodium hydrate drug combination," "LCZ696," "angiotensin receptor/neprilysin inhibit," "ARNI," "neprilysin inhibit," "randomized controlled trial," "RCT," "controlled clinical trial," "random," "placebo," and "trial." To find relevant articles, these keywords were combined in varying combinations using the Booleans "and," "OR," and "not."

Timeline of the Study

The literature search was carried out to identify studies conducted from 1 January 2010 to 1 January 2023.

Study Selection

Inclusion criteria: The inclusion criteria included the randomized controlled trials that included adult patients aged 18 and above with heart failure with reduced ejection fraction (HFrEF).

Exclusion criteria: The exclusion criteria included duplicate reports, studies based on patients having HFpEF, studies that did not provide enough data to analyze the primary outcome, and studies in which the full text was not available.

Data Extraction Method

The data for this research project was extracted from each included study using a standardized data collection form that included the following: trial number/identifier, author, publication year, demographic characteristics, number of patients, intervention, control treatment, HRQoL outcome characteristics (scales and change from baseline), and follow-up duration. The data analysis was performed by using the software RevMan 5.4 (Cochrane Collaboration, London, United Kingdom) [38].

The Statistical Method Used for the Analysis

Since different scales and questionnaires were used to assess the HRQoL, the continuous outcomes are presented as standardized mean difference (SMD) with 95% confidence intervals (CIs). For dichotomous outcomes, data were presented as risk ratio (RR) with 95% confidence intervals (CIs). The Z-test was used to calculate whether the pooled effect was statistically significant (p < 0.05). The χ^2^ test was used to test heterogeneity. If there was no significant heterogeneity among the studies (p ≥ 0.10; I^2^ < 50%), the fixed-effects model was sought. If the heterogeneity among the studies was large (p < 0.10; I^2^ ≥ 50%), the random effects model sought and analyzed the causes of heterogeneity [39]. In the event of unexplained heterogeneity, a descriptive analysis of the findings from each study was carried out.

Risk of Bias Assessment

The risk of bias in the included studies was assessed using the Cochrane Collaboration Risk of Bias tool, and the assessment contents included (1) random sequence generation, (2) allocation concealment, (3) blinding of subjects and researchers, (4) blinding of outcome measurement, (5) incomplete outcome data, (6) selective outcome reporting, and (7) other biases [40].

Quality of Evidence Assessment

The quality of evidence for the primary outcome was done using the Grading of Recommendations, Assessment, Development, and Evaluation (GRADE) framework [41], which contains five downgrading factors: study limitation, inconsistency, indirectness, imprecision, and publication bias [42].

Results

Study Selection

The systemic search using the aforementioned parameters resulted in a total of 458 studies. Out of these 458 studies derived from the search, a total of eight studies [43-50] were finally included in this review. The details of the studies included in this systematic review and meta-analysis are shown below.

The ACTIVITY-HF trial [43] investigated the brief effects of sacubitril/valsartan on the strong counterpart enalapril in enhancing high-intensity exercise ability in individuals with heart failure and a low ejection fraction. It was a randomized, double-blinded, socially engaged study.

In the AWAKE-HF study [44], actigraphy was used to compare the effects of starting sacubitril/valsartan against enalapril on activity and sleep in patients with heart failure and a low ejection fraction.

The Effect of Sacubitril-Valsartan vs Enalapril on Aortic Stiffness in Patients With Heart Failure and Reduced Ejection Fraction (EVALUATE-HF) [45] examined that, compared to enalapril, sacubitril/valsartan has therapeutic effects that are partly mediated by pathophysiological processes in individuals with heart failure and a low ejection fraction. This trial went on to ascertain if sacubitril/valsartan therapy for HFrEF improves central aortic stiffness and cardiac remodeling as opposed to enalapril.

The OUTSTEP-HF trial [46] evaluated the impact of sacubitril/valsartan versus enalapril on individuals with heart failure (HF) and a diminished ejection fraction complaint, six-minute walk test (6MWT) distances, and non-sedentary daily physical activity.

The Prospective Comparison of ARNi With ACE-I to Determine Impact on Global Mortality and Morbidity in Heart Failure (PARADIGM-HF) trial [47] examined the effects of enalapril and the angiotensin receptor-neprilysin inhibitor in individuals with cardiovascular disease with a low ejection fraction. The experiment was intended to find a disparity in the mortality rates through circulatory diseases, although the primary endpoint was a combination of death from cardiovascular causes or hospitalization for heart problems.

The Prospective comparison of ARNI with ACE inhibitor to determine the noveL beneficiaL trEatment vaLue in Japanese Heart Failure (PARALLEL-HF) trial [48] was carried out to examine the performance and safety of sacubitril/valsartan in Japanese HFrEF individuals; a prospective randomized experiment was carried out.

A study in Brazil comprised 52 patients with HFrEF and a left ventricular ejection fraction of less than 40% who were given enalapril or sacubitril/valsartan [49]. Through the use of aerobic endurance training, peak oxygen consumption (VO_2_) was determined. A six-minute stroll trial was also conducted in this study.

**Figure 1:**
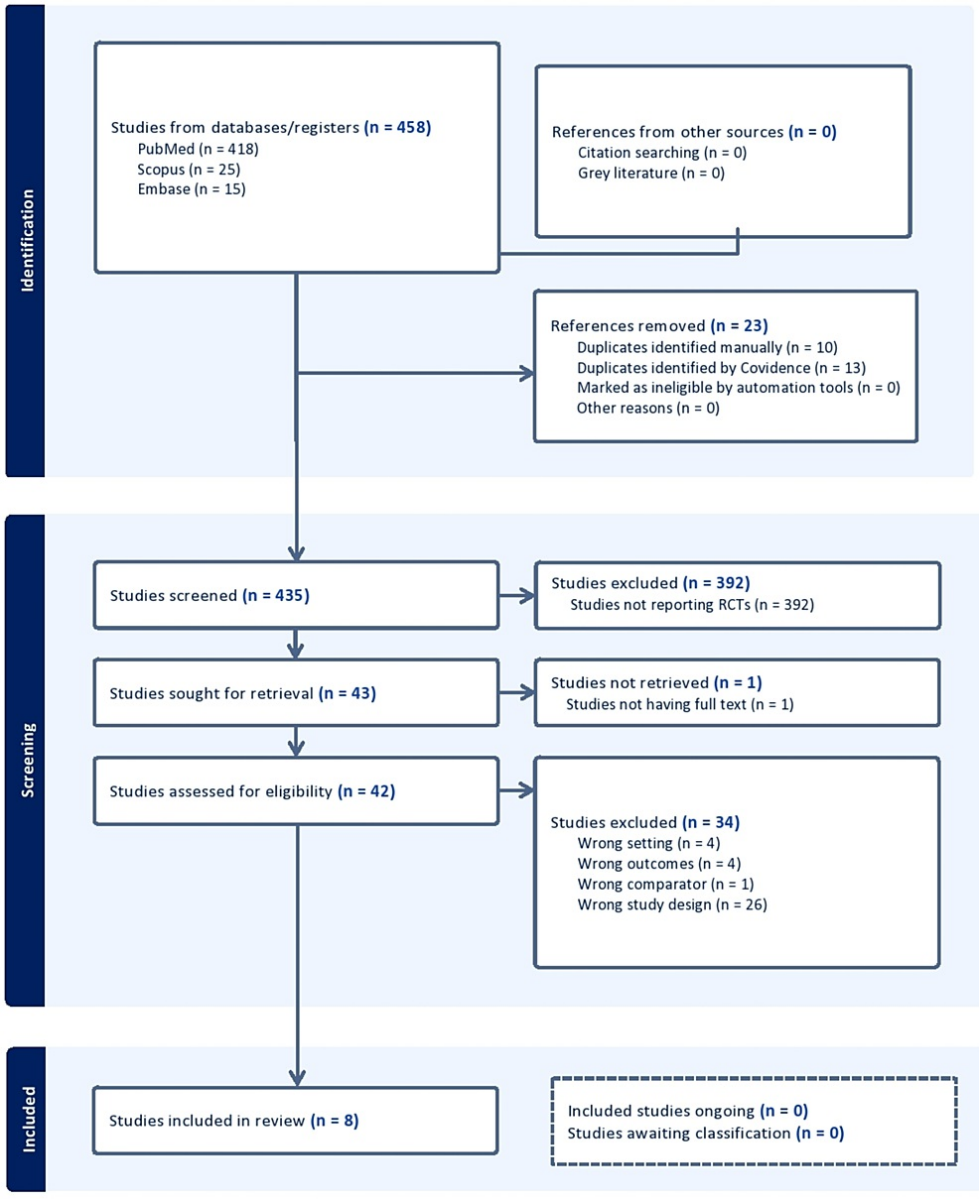
The PRISMA flowchart used for the study selection PRISMA, Preferred Reporting Items for Systematic Reviews and Meta-Analyses; RCTs, randomized clinical trials

The LCZ696 In Hospitalized Advanced Heart Failure (LIFE-HF) study [50] dealt with sacubitril/valsartan's stability and acceptability in people with advanced chronic heart failure. This study was conducted to contrast valsartan treatment with sacubitril management in individuals with symptomatic heart failure, a low ejection fraction, and current class IV symptoms according to the New York Heart Association. The present study followed Preferred Reporting Items for Systematic Reviews and Meta-Analyses (PRISMA) guidelines (Figure 1) [51].

The metadata obtained from the analysis of these selected eight studies is summarized in the tables below along with their detailed description of the analysis.

Baseline Characteristics of the Included Studies

Over the course of a decade, the included trials evaluated the drug sacubitril/valsartan against a control drug to check for its efficacy, safety, and treatment modality to ensure HF patients achieve a stable quality of life. Table 1 consists of the baseline characteristics of the selected studies with respect to the sample sizes of the experimental drug and control drug; it also lists the "study ID," which are the trial registration numbers of the individual trials, and the year they were conducted.

**Table 1:** Analysis of the baseline characteristics of the selected studies with respect to the sample sizes of the experimental drug and control drug EVALUATE-HF, Effect of Sacubitril-Valsartan vs Enalapril on Aortic Stiffness in Patients With Heart Failure and Reduced Ejection Fraction; LIFE-HF, LCZ696 In Hospitalized Advanced Heart Failure; PARADIGM-HF, Prospective Comparison of ARNi With ACE-I to Determine Impact on Global Mortality and Morbidity in Heart Failure; PARALLEL-HF, Prospective comparison of ARNI with ACE inhibitor to determine the noveL beneficiaL trEatment vaLue in Japanese Heart Failure

Study ID	Name of trial/author	Year	Sample size	
			Sacubitril/valsartan	Control
NCT02768298	ACTIVITY-HF [43]	2021	103	98
NCT02970669	AWAKE-HF [44]	2021	70	70
NCT02874794	EVALUATE-HF [45]	2019	231	233
NCT02816736	LIFE-HF [50]	2022	167	168
NCT02900378	OUTSTEP-HF [46]	2021	309	310
NCT01035255	PARADIGM-HF [47]	2014	4209	4233
NCT02468232	PARALLEL-HF [48]	2021	112	113
NCT03190304	Dos Santos et al. [49]	2021	26	18

Comparison of the Age Groups Across the Included Trials

HF is a systemic disease rather than a singular phenomenon, and its symptoms might vary depending on factors such as age, gender, race, ethnicity, LVEF status, and the cause of the condition. Additionally, patients with HF and reduced LVEF exhibit different metabolic characteristics from those with HF, as well as intact LVEF, which recent investigations are starting to recognize deeper [52]. The existing literature has demonstrated that the mortality rate in HF patients is age-dependent and gradually rises with aging [53,54]. The Global Health Data Exchange registry also records a racial preference, with individuals of African American heritage having a 25% greater prevalence of heart failure than Caucasians. According to the American Heart Association, heart failure continues to be the leading reason for older patients to be hospitalized and is to blame for the 8.5% of cardiorespiratory-related fatalities in the United States [8]. According to this same study, heart failure is more common and has greater incidence rates among African Americans, Hispanic Americans, Native Americans, and recent immigrants from developing countries [8]. The prevalent rate of chronic HF is comparatively higher in younger individuals, which again was related to metabolic syndrome as the etiology, according to the Candesartan in Heart Failure Assessment of Reduction in Mortality and Morbidity (CHARM) program [55]. The average age of patients at the time of death rose over the past 10 years as life expectancy in Western countries grew, going from 70 to 81 years before 1980 [54]. Following the age of 65, the rate of the occurrence of heart failure in males doubles with every 10 years of age rise, while it skyrockets in females for the same age bracket [8].

According to Saczynski et al., inhospital fatality rates rose from 3% for individuals under the age of 65 to 8.2% for some over the age of 75 [56]. The current systematic review exhibits that the included trials had a wide range of individuals coming from different age groups. The mean and standard deviation have been calculated for each age group in the respective clinical trials. In Table 2, a comparison has been done with respect to the age of the patients receiving the drugs (control drug versus trial drug) in the respective trials.

**Table 2:** Analysis of the selected trials with respect to age distribution among the trial drug and control drug SD, standard deviation; EVALUATE-HF, Effect of Sacubitril-Valsartan vs Enalapril on Aortic Stiffness in Patients With Heart Failure and Reduced Ejection Fraction; LIFE-HF, LCZ696 In Hospitalized Advanced Heart Failure; PARADIGM-HF, Prospective Comparison of ARNi With ACE-I to Determine Impact on Global Mortality and Morbidity in Heart Failure; PARALLEL-HF, Prospective comparison of ARNI with ACE inhibitor to determine the noveL beneficiaL trEatment vaLue in Japanese Heart Failure

Study ID	Name of trial/author	Year	Age (mean ± SD)	
			Sacubitril/valsartan	Control
NCT02768298	ACTIVITY-HF [43]	2021	66.1 ± 10.8	67.6 ± 10.0
NCT02970669	AWAKE-HF [44]	2021	62.3 ± 8.8	64.2 ± 11.6
NCT02874794	EVALUATE-HF [45]	2019	67.8 ± 9.8	66.7 ± 8.5
NCT02816736	LIFE-HF [50]	2022	60.2 ± 13.4	58.3 ± 13.1
NCT02900378	OUTSTEP-HF [46]	2021	67.16 ± 11.04	66.62 ± 10.45
NCT01035255	PARADIGM-HF [47]	2014	63.78 ± 11.52	63.82 ± 11.25
NCT02468232	PARALLEL-HF [48]	2021	69.0 ± 9.7	66.7 ± 10.9
NCT03190304	Dos Santos et al. [49]	2021	55.4 ± 3.34	59.14 ± 4.23

**Figure 2:**
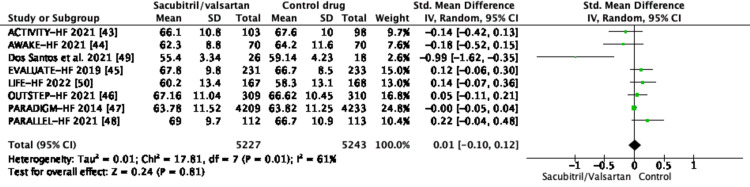
Forest plot of the comparison of the selected trials with respect to age distribution among the trial drug and control drug SD, standard deviation; CI, confidence interval; IV, inverse variance; df, degrees of freedom; EVALUATE-HF, Effect of Sacubitril-Valsartan vs Enalapril on Aortic Stiffness in Patients With Heart Failure and Reduced Ejection Fraction; LIFE-HF, LCZ696 In Hospitalized Advanced Heart Failure; PARADIGM-HF, Prospective Comparison of ARNi With ACE-I to Determine Impact on Global Mortality and Morbidity in Heart Failure; PARALLEL-HF, Prospective comparison of ARNI with ACE inhibitor to determine the noveL beneficiaL trEatment vaLue in Japanese Heart Failure

The forest plot of the above comparison (Figure 2) shows that the I^2^ value is 61% (i.e., I^2^ > 50%), which relays that there is substantial heterogeneity within the studies.

Comparison of the Drug Dosage Used Between Trials

When the doses of the drugs were looked at, sacubitril/valsartan had a standard of 200 mg twice daily (BD) dose. The control drug was enalapril in most studies (barring the LIFE-HF trial), with the dose being 10 mg BD; however, on the study done in Brazil, it was 20 mg BD [49]. The LIFE-HF trial used valsartan as a control and had a dosage of 160 mg BD (Table 3).

**Table 3:** Analysis of the dosage of the trial drug and the control drug BD, twice daily; EVALUATE-HF, Effect of Sacubitril-Valsartan vs Enalapril on Aortic Stiffness in Patients With Heart Failure and Reduced Ejection Fraction; LIFE-HF, LCZ696 In Hospitalized Advanced Heart Failure; PARADIGM-HF, Prospective Comparison of ARNi With ACE-I to Determine Impact on Global Mortality and Morbidity in Heart Failure; PARALLEL-HF, Prospective comparison of ARNI with ACE inhibitor to determine the noveL beneficiaL trEatment vaLue in Japanese Heart Failure

Study ID	Name of trial/author	Year	Intervention			
			Drug randomized	Dose	Control	Dose
NCT02768298	ACTIVITY-HF [43]	2021	Sacubitril/valsartan	200 mg BD	Enalapril	10 mg BD
NCT02970669	AWAKE-HF [44]	2021	Sacubitril/valsartan	200 mg BD	Enalapril	10 mg BD
NCT02874794	EVALUATE-HF [45]	2019	Sacubitril/valsartan	200 mg BD	Enalapril	10 mg BD
NCT02816736	LIFE-HF [50]	2022	Sacubitril/valsartan	200 mg BD	Valsartan	160 mg BD
NCT02900378	OUTSTEP-HF [46]	2021	Sacubitril/valsartan	200 mg BD	Enalapril	10 mg BD
NCT01035255	PARADIGM-HF [47]	2014	Sacubitril/valsartan	200 mg BD	Enalapril	10 mg BD
NCT02468232	PARALLEL-HF [48]	2021	Sacubitril/valsartan	200 mg BD	Enalapril	10 mg BD
NCT03190304	Dos Santos et al. [49]	2021	Sacubitril/valsartan	200 mg BD	Enalapril	20 mg BD

Comparison of the NYHA Classification of the Study Participants

In the PARADIGM-HF trial [47], all the predetermined subgroups saw the same effects with LCZ696. The interaction between NYHA class at randomization and the effect of the treatment on the major endpoint did not reach statistical significance (p = 0.76) nor for the effect of the drug on mortality from cardiovascular causes (p = 0.03, without multiple comparison adjustments). In the OUTSTEP-HF study [46], a logistic regression strategy with the odds ratio (OR), the initial value, the therapy, and the baseline NYHA class as fixed factors was followed.

When compared to the enalapril group, in the PARALLEL-HF study [48], the sacubitril/valsartan group had numerically more patients who had improved their NYHA functional class from randomization to week 8 (13.5% versus 10.8%). The NYHA functional class progress between the treatment arms did not vary significantly altogether at the predetermined time intervals (week 4, week 8, month 6, and last assessment: p = 0.7115, p = 0.1752, p = 0.2688, and p = 0.5798, respectively). Remarkably, at week 4, week 8, week 12, month 6, and last assessment, the proportion of patients whose NYHA functional class decreased from baseline was lower in the sacubitril/valsartan group than in the enalapril group (0.9% versus 4.5%), and it was also lower at month 6 (5.4% versus 9.0%) [48]. In the EVALUATE-HF trial [45], 67.4% of the included subjects had NYHA class II functional status, and at follow-up, the change was insignificant to compare (Table 4).

**Table 4:** Analysis of the trials comparing the NYHA classification of the patients included during the trial NYHA, New York Heart Association; EVALUATE-HF, Effect of Sacubitril-Valsartan vs Enalapril on Aortic Stiffness in Patients With Heart Failure and Reduced Ejection Fraction; LIFE-HF, LCZ696 In Hospitalized Advanced Heart Failure; PARADIGM-HF, Prospective Comparison of ARNi With ACE-I to Determine Impact on Global Mortality and Morbidity in Heart Failure; PARALLEL-HF, Prospective comparison of ARNI with ACE inhibitor to determine the noveL beneficiaL trEatment vaLue in Japanese Heart Failure

Study ID	Name of trial/author	Year	NYHA class II/III/IV, %		
			NYHA II	NYHA III	NYHA IV
NCT02768298	ACTIVITY-HF [43]	2021	0.5	99.5	0
NCT02970669	AWAKE-HF [44]	2021	90	10	0
NCT02874794	EVALUATE-HF [45]	2019	67.5	21.6	0
NCT02816736	LIFE-HF [50]	2022	22.4	40.09	34
NCT02900378	OUTSTEP-HF [46]	2021	52.2	47.2	0.7
NCT01035255	PARADIGM-HF [47]	2014	70.4	24	0.7
NCT02468232	PARALLEL-HF [48]	2021	92.04	4.5	0
NCT03190304	Dos Santos et al. [49]	2021	53.8	46.2	0

Comparison of the Mean LVEF (Percentage) of the Study Samples

A good prognosis has indeed been factored into individuals with HF with low EF who recoup their LVEF in previous studies [57]. But this trial that studied the effects of valsartan, at a 12-month follow-up, too could not provide significant improvement of the LVEF of the subgroup having HFrEF. The favorable outcome was noticed only in a minority of patients [57]. Hence, it was imperative to check if the trials included in this study had measured improvement in LVEF after the trial drug Entresto had been administered. The mean left ventricular ejection function (LVEF) in percentage is listed in the following table. Of all the trials included in the systematic review, OUTSTEP-HF did not evaluate the LVEF (Table 5).

**Table 5:** Analysis of the trials comparing the mean LVEF (percentage) of the patients having HFrEF HFrEF, heart failure with reduced ejection fraction; LVEF, left ventricular ejection fraction; N/A, not available; EVALUATE-HF, Effect of Sacubitril-Valsartan vs Enalapril on Aortic Stiffness in Patients With Heart Failure and Reduced Ejection Fraction; LIFE-HF, LCZ696 In Hospitalized Advanced Heart Failure; PARADIGM-HF, Prospective Comparison of ARNi With ACE-I to Determine Impact on Global Mortality and Morbidity in Heart Failure; PARALLEL-HF, Prospective comparison of ARNI with ACE inhibitor to determine the noveL beneficiaL trEatment vaLue in Japanese Heart Failure

Study ID	Name of trial/author	Year	Mean LVEF (%)
NCT02768298	ACTIVITY-HF [43]	2021	31.9
NCT02970669	AWAKE-HF [44]	2021	30.9
NCT02874794	EVALUATE-HF [45]	2019	33.5
NCT02816736	LIFE-HF [50]	2022	20.04
NCT02900378	OUTSTEP-HF [46]	2021	N/A
NCT01035255	PARADIGM-HF [47]	2014	29.5
NCT02468232	PARALLEL-HF [48]	2021	28.1
NCT03190304	Dos Santos et al. [49]	2021	26

Subgroup Analysis of LVEF

**Figure 3:**
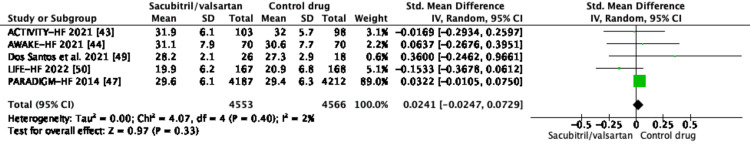
Subgroup analysis of trials according to LVEF LVEF, left ventricular ejection fraction; SD, standard deviation; CI, confidence interval; IV, inverse variance; df, degrees of freedom; LIFE-HF, LCZ696 In Hospitalized Advanced Heart Failure; PARADIGM-HF, Prospective Comparison of ARNi With ACE-I to Determine Impact on Global Mortality and Morbidity in Heart Failure

A subgroup analysis of comparable parameters was done wherever feasible. A heterogeneity test was performed using the random effects model. Left ventricular ejection fraction (LVEF) was reported in the five studies as depicted in Figure 3, and hence, a subgroup analysis that yielded a confidence interval (CI) of 95% had a standard mean difference of 0.02 (-0.02, 0.07). The forest plot depicts barely any noticeable significance of sacubitril/valsartan versus the control drug over LVEF.

There were not enough data to clearly demarcate if LVEF changes were significantly noticeable at follow-up in the individual trials. It is an important factor to note that a previous statistically sound study [58] that tried to evaluate the heterogeneity of outcomes among HF patients with ventricular recovery found that their quoted "best outcome" (which was essentially LVEF rising from 35% at baseline to >40% at three or 12 months in accordance with the LVEF limits used to direct the implantation of primary preventive implantable cardioverter-defibrillators {ICDs}) consists of a diverse patient population with differences in both the stability and timing of left ventricular restoration. Crucially, these results include both right and left ventricular diastolic function, indicating that improving LVEF can serve as a proxy for complete myocardial recovery [58]. However, they evaluated the aftermath of ARBs and ACE inhibitors.

Analysis of the Levels of NT-proBNP and HFrEF

In order to assess changes in left ventricular ejection fraction (LVEF) in patients with heart failure and lower LVEF managed with sacubitril/valsartan, a prospective, single-armed, observation cohort research was conducted in Taiwan [59]. Independent of whether a subject was currently receiving a standard prescription or had just received a diagnosis of HFrEF, the researchers recommended sacubitril/valsartan as both first-line and second-line therapy to all eligible patients [59]. In this Taiwanese cohort, sacubitril/valsartan showed a significant improvement in LVEF and left ventricular reverse remodeling and a decrease in NT-proBNP [59]. It was deemed necessary to check if similar effects were noticed in the included double-blinded RCTs. Due to the difference in the follow-up duration of the trials, a descriptive analysis of the levels of NT-proBNP across the trials is done.

According to the LIFE-HF study findings, reducing NT-proBNP levels in patients with advanced heart failure did not differ between sacubitril/valsartan and valsartan. This result was true for the majority of the subgroups investigated. By week 24 of treatment, the geometric mean NT-proBNP level in both the sacubitril/valsartan and valsartan treatment groups had fallen below baseline levels [50]. The geometric mean NT-proBNP level remained elevated over the course of eight weeks of therapy. The area under the curve (AUC) of NT-proBNP levels at two, four, eight, 12, and 24 weeks compared with the level of NT-proBNP at randomization served as the primary efficacy outcome. The NT-proBNP concentrations recorded at 2-24 weeks were divided by the concentration at randomization to determine the endpoint. The trapezoidal rule was used to calculate the AUC for the ratio of NT-proBNP relative to baseline through 24 weeks of medication [50]. The median AUC for NT-proBNP in the sacubitril/valsartan treatment arm was 1.08 (interquartile range {IQR}: 0.75-1.60), and in the valsartan treatment arm, it was 1.19 (IQR: 0.91-1.64). Sacubitril/valsartan versus valsartan had an estimated ratio of change for the AUC (primary endpoint) of 0.95 (95% CI: 0.84, 1.08; p = 0.45) [50].

In the EVALUATE-HF trial, at 12 weeks, the levels of NT-proBNP decreased more in the sacubitril/valsartan group than in the enalapril group [45]. The PARADIGM-HF trial enrolled patients with at least mildly elevated natriuretic peptide levels in an effort to match their anticipated event rate, but the attributes of their patients with heart failure were comparable to study populaces in other pertinent trials at the time, as well as patients in the public at large [47,60,61].

The PARALLEL-HF trial done in the Japanese cohort demonstrated that patients in the sacubitril/valsartan and enalapril groups experienced reductions in plasma NT-proBNP levels of 21.3% and 22.9%, respectively, after receiving treatment with 50 mg of sacubitril/valsartan twice daily for two weeks during the run-in phase. After that, NT-proBNP levels were lowered by 27.1% from the run-in baseline after two weeks of treatment with sacubitril/valsartan 100 mg BD post randomization, compared to 1.9% with enalapril 5 mg BD (i.e., 20% increase in NT-proBNP levels post randomization) [48]. Sacubitril/valsartan drastically decreased NT-proBNP levels more than enalapril following four weeks of treatment post randomization (23.3% versus 11.4%). Up until six months after the randomization, treatment with sacubitril/valsartan 200 mg BD decreased NT-proBNP by 30.5% from the run-in benchmark as opposed to a 14.4% decline with enalapril 10 mg BD. A statistically considerable reduction in NT-proBNP was seen in the sacubitril/valsartan group compared to the enalapril subgroup as early as week 2 (between-group difference in percentage reduction, 25.7%; p = 0.0001) [48]. There were 13.4% and 14.6% between-group differences in the percent decreases of NT-proBNP at weeks 4 and 8, respectively (least square means {LSM} of ratio: 0.87; 95% CI: 0.76, 0.99; p = 0.0326; LSM of ratio: 0.85; 95% CI: 0.75, 0.97; p = 0.0161) [48]. Through month 6, there was an 18.9% difference in the decreases between the two groups due to sacubitril/valsartan's superior effect when compared to enalapril (LSM of ratio: 0.81; 95% CI: 0.69, 0.95; p = 0.0104) [48].

The study done in Brazil showed that BNP and NT-proBNP levels did not change substantially after 12 weeks (sacubitril/valsartan: from 118.0 {76.5-421.5} to 147 {80.0-546} pg/mL and enalapril: from 272.0 {67.8-1269.8} to 182.0 {59.8-932.3} pg/mL; p = 0.358 and p = 0.125, respectively) [49].

Comparison of the Follow-Up Duration of the Trials

Table 6 shows the individual follow-up duration of the trials in weeks. As they were not exactly similar in duration and data were not consistently available for the duration that overlapped, a descriptive analysis has been done and summarized in Table 6. The ACTIVITY-HF trial had a follow-up duration of 12 weeks, while the AWAKE-HF trial had a follow-up period of eight weeks. While the EVALUATE-HF trial had a 12-week follow-up period, it is seen that the LIFE-HF trial had a 24-week follow-up duration. The OUTSTEP-HF trial had a follow-up of 12 weeks, whereas the PARADIGM-HF had a follow-up period of 32 weeks.

**Table 6:** Analysis of the trials comparing follow-up duration EVALUATE-HF, Effect of Sacubitril-Valsartan vs Enalapril on Aortic Stiffness in Patients With Heart Failure and Reduced Ejection Fraction; LIFE-HF, LCZ696 In Hospitalized Advanced Heart Failure; PARADIGM-HF, Prospective Comparison of ARNi With ACE-I to Determine Impact on Global Mortality and Morbidity in Heart Failure; PARALLEL-HF, Prospective comparison of ARNI with ACE inhibitor to determine the noveL beneficiaL trEatment vaLue in Japanese Heart Failure

Study ID	Name of trial/author	Year	Follow-up duration
NCT02768298	ACTIVITY-HF [43]	2021	12 weeks
NCT02970669	AWAKE-HF [44]	2021	8 weeks
NCT02874794	EVALUATE-HF [45]	2019	12 weeks
NCT02816736	LIFE-HF [50]	2022	24 weeks
NCT02900378	OUTSTEP-HF [46]	2021	12 weeks
NCT01035255	PARADIGM-HF [47]	2014	32 weeks
NCT02468232	PARALLEL-HF [48]	2021	24 weeks
NCT03190304	Dos Santos et al. [49]	2021	24 weeks

Comparison of the Tools Used for Health-Related Quality of Life (HRQoL) Measurements

The HRQoL scales are approved instruments for evaluating individuals' standard of health. It can be separated into universal scales and specialized dimensions based on various testing goals. The Kansas City Cardiomyopathy Questionnaire (KCCQ) and the Minnesota Living with Heart Failure Questionnaire (MLHFQ) are internationally acclaimed cardiovascular failure-specific HRQoL scales, whereas the Short Form 36 (SF-36) and Nottingham Health Profile (NHP) are universal scales [62].

**Figure 4:**
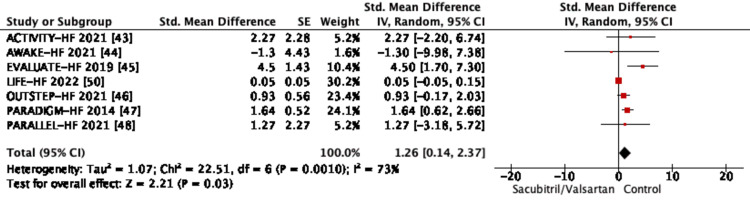
Subgroup analysis of trials reporting health-related quality of life (HRQoL) SE, standard error; CI, confidence interval; IV, inverse variance; df, degrees of freedom; EVALUATE-HF, Effect of Sacubitril-Valsartan vs Enalapril on Aortic Stiffness in Patients With Heart Failure and Reduced Ejection Fraction; LIFE-HF, LCZ696 In Hospitalized Advanced Heart Failure; PARADIGM-HF, Prospective Comparison of ARNi With ACE-I to Determine Impact on Global Mortality and Morbidity in Heart Failure; PARALLEL-HF, Prospective comparison of ARNI with ACE inhibitor to determine the noveL beneficiaL trEatment vaLue in Japanese Heart Failure

Since the same tool was not uniformly used in all the trials (Table 7), a meta-analysis was not feasible for all the included studies; hence, the Brazilian study has been excluded, and a descriptive analysis has been done. This is summarized in Figure 4.

**Table 7:** Analysis of the trials comparing the tools used for health-related quality of life (HRQoL) measurements KCCQ OSS, Kansas City Cardiomyopathy Questionnaire overall summary score; SF-12, Short Form 12; EQ-SD-5L, EuroQol 5 Dimension 5 Level; EVALUATE-HF, Effect of Sacubitril-Valsartan vs Enalapril on Aortic Stiffness in Patients With Heart Failure and Reduced Ejection Fraction; LIFE-HF, LCZ696 In Hospitalized Advanced Heart Failure; PARADIGM-HF, Prospective Comparison of ARNi With ACE-I to Determine Impact on Global Mortality and Morbidity in Heart Failure; PARALLEL-HF, Prospective comparison of ARNI with ACE inhibitor to determine the noveL beneficiaL trEatment vaLue in Japanese Heart Failure

Study ID	Name of trial/author	Year	HRQoL tool
NCT02768298	ACTIVITY-HF [43]	2021	KCCQ OSS
NCT02970669	AWAKE-HF [44]	2021	KCCQ OSS
NCT02874794	EVALUATE-HF [45]	2019	KCCQ OSS
NCT02816736	LIFE-HF [50]	2022	KCCQ OSS
NCT02900378	OUTSTEP-HF [46]	2021	SF-12 physical function subscale and EQ-SD-5L
NCT01035255	PARADIGM-HF [47]	2014	KCCQ OSS
NCT02468232	PARALLEL-HF [48]	2021	KCCQ OSS
NCT03190304	Dos Santos et al. [49]	2021	Minnesota Living with Heart Failure Questionnaire

According to the AWAKE-HF trial, in about week 8, there were almost no considerable variations in the geometric mean ratio of activity counts during the busiest 30 minutes of the day (0.9456 {sacubitril/valsartan/enalapril}; 95% confidence interval {CI}: 0.8863, 1.0088; p = 0.0895) or in the mean change from baseline in activity during sleep (difference: 2.038 counts/minute; 95% CI: -0.062, 4.138; p = 0.0570). Sacubitril/valsartan showed a sub-chronic change from baseline to week 8 on the KCCQ-23 (2.89 for sacubitril/valsartan and 4.19 for enalapril).

As per the EVALUATE-HF trial, in the sacubitril/valsartan pool, the KCCQ overall summary score (OSS) increased by 8.9 points, while in the enalapril group, it decreased by 4.3 points (between-group difference: 4.5 points; 95% CI: 1.7, 7.3; p = 0.002) [45]. Premised on a repeated measures analysis of covariance model, the change from baseline in the overall summary score and component scores was examined. Treatment, week, and treatment-week interaction were included as fixed-effects factors and baseline value as a covariate, with a common unstructured covariance for each treatment group [45]. The percent of participants in the sacubitril/valsartan group who saw an improvement of five points or more in their KCCQ overall summary score (KCCQ OSS) was also greater (58% versus 43%; p = 0.001). A post hoc analysis revealed a correlation between enhancements in NT-proBNP and changes in quality of life [45].

From the PARALLEL-HF study's randomization to the pre-specified time points of week 8 (LSM change from baseline for sacubitril/valsartan versus enalapril: 0.05 versus 2.59; p = 0.1854) and month 6 (2.22 versus 3.49; p = 0.5737), sacubitril/valsartan showed a trend to a lesser deterioration of KCCQ clinical summary score for HF symptoms and physical limitations [48]. The KCCQ clinical summary score in the PARADIGM-HF trial decreased on average by 2.99 points in the LCZ696 group and 4.63 points in the enalapril group between baseline and month 8 (between-group difference: 1.64 points; 95% confidence interval: 0.63, 2.65; p = 0.001).

A subgroup analysis was performed using the pooled effect value by random effects model. The findings showed that the sacubitril/valsartan group significantly outperformed the control group in terms of improving HFrEF HRQoL (SMD: 1.26; 95% CI: 0.14, 2.37; p = 0.03) as shown in Figure 4.

Comparison of 6MWT, VO_2_, Physical Activity, and Exercise Capacity

The trials had disparity between reporting of effect on VO_2_, 6MWT, physical activity, and exercise capacity. Hence, a descriptive analysis was performed on those trials reporting these outcomes. In the ACTIVITY-HF trial, the peak VO_2_ was measured by cardiopulmonary exercise testing (CPET) using a cycle ergometer during screening throughout the week, and week 6 and week 12 visits in accordance with most materials have emerged [43].

At week 6 and week 12, there were no discernible treatment variations for alterations in the ventilation/volume of exhaled carbon dioxide (VE/VCO_2_) slope. The Borg scale for dyspnea and exhaustion showed a similar change in the rate of perceived exertion during exercise in both treatment groups at baseline and at week 12, showing that the patients in both arms of the study similarly fatigued themselves during CPET [43].

In patients with heart failure (HF) with a decreased ejection fraction, OUTSTEP-HF examined the impact of sacubitril/valsartan versus enalapril on a six-minute walk test (6MWT) distance, non-sedentary daytime physical activity, and HFrEF symptoms [46]. Sacubitril/valsartan (n = 310) or enalapril (n = 311) were randomly assigned to ambulatory individuals (n = 621) with stable symptoms of HFrEF. Using the 6MWT and an accelerometer worn on the wrist, changes in physical activity and mean daily non-sedentary daytime activity were assessed from baseline to week 12. There was no statistically significant variation (least square mean treatment difference: 8.98 m; 97.5% CI: 1.31, 19.27; p = 0.0503) after the 6MWT improved by 35.09 m with sacubitril/valsartan and by 26.11 m with enalapril after 12 weeks [46]. After 12 weeks, the daily average non-sedentary performance degrades with sacubitril/valsartan by 27 minutes and with enalapril by 21 minutes (least square mean treatment difference: six minutes; 97.5% CI: 25.7, 13.4; p = 0.4769). Fifty-one percent of patients receiving sacubitril/valsartan saw an improvement in their 6MWT of less than 30 m, compared to 44% of people receiving enalapril (odds ratio: 1.251; 95% CI: 0.895, 1.748) [46]. At week 4, 58% of patients receiving sacubitril/valsartan experienced an increase in non-sedentary daytime activity compared to 64% of patients receiving enalapril, and 58% of patients receiving sacubitril/valsartan reported improved HF symptoms as measured by the patient global assessment compared to 43% of patients receiving enalapril. But by week 12, these discrepancies had disappeared. In summary, the OUTSTEP-HF found no statistically significant difference between sacubitril/valsartan medication and enalapril in terms of physical activity in patients with heart failure and reduced ejection fraction [46].

The RCT done in Brazil [49] examined the effects of enalapril and sacubitril/valsartan in individuals with HFrEF using peak oxygen consumption (VO_2_) and the six-minute walk test (6MWT). This trial recruited 52 patients with HFrEF and a left ventricular ejection fraction of less than 40% who were randomly assigned to receive either enalapril or sacubitril/valsartan. Through the use of cardiopulmonary exercise training, peak VO_2_ was determined. A test of a six-minute walk has also been conducted. At 12 weeks, peak VO_2_ climbed by 13.1% (19.35 ± 0.99 to 21.89 ± 1.04 mL/kg/minute) in the sacubitril/valsartan group (mean dose: 382.6 ± 57.6 mg daily) and by 5.6% (18.58 ± 1.19 to 19.62 ± 1.25 mL/kg/minute ) in the enalapril cohort (mean dose 34.4 ± 9.2 mg daily). However, there was no distinction between the groups (p = 0.332 interaction). Peak VO_2_ rose in both sacubitril/valsartan (mean dose: 400 ± 0 mg daily) and enalapril (mean dose: 32.7 ± 11.0 mg daily) at 24 weeks by 13.5% (19.35 ± 0.99 to 21.96 ± 0.98 mL/kg/minute) and 12.0% (18.58 ± 1.19 to 20.82 ± 1.18 mL/kg/minute), correspondingly. Following 12 or 24 weeks, individuals with HFrEF showed no appreciable improvement in peak VO_2_ or 6MWT as contrasted to enalapril [49].

Discussion

According to recent scientific literature, patients with heart failure statistically significantly worsen in all areas of quality of life, not only in physical functionality. Compared to other very common chronic illnesses, whether cardiac or affecting other systems, the physical (role and functioning) health burden was much greater. Patients with heart failure appear to perceive their quality of life as being better when their treatment is optimized to enhance their NYHA class. Given the sharp deterioration in the quality of life associated with heart failure, healthcare interventions should focus considerably more on this endpoint, especially those that use medications such as ACE inhibitors and beta-blockers, which have been demonstrated to enhance the quality of life [35].

In the current systematic review, it is important to note that in the PARADIGM-HF clinical study, sacubitril/valsartan, a first-in-class angiotensin receptor-neprilysin inhibitor (ARNI), demonstrated primacy over enalapril in lowering cardiac deaths and readmission [47].

Only the NYHA functional class was consistently and closely related to all quality-of-life scores, according to a study's multiple regression analysis. Only one of the eight quality-of-life areas (physical functioning) was explained by the six-minute walk test and peak oxygen consumption records [36].

Along with other clinical outcomes, sacubitril/valsartan is also known to enhance health-related quality of life (HRQoL) and symptom load [63]. Sacubitril/valsartan markedly increased reactions in sexual and domestic activities in patients with heart failure and low ejection fraction. In addition to lowering the risk of cardiovascular death, all-cause mortality, and hospitalization for heart failure, sacubitril/valsartan may help these patients with their restrictions in daily activities [64,65]. A shift of 30-35 m in the 6MWT is regarded as clinically significant [66,67].

Through week 12, sacubitril/valsartan treatment in ACTIVITY-HF increased the 6MWT by 29 m as opposed to 17 m with enalapril, but the exploratory endpoint showed no statistically significant changes between the treatment groups. These findings are consistent with the previously released OUTSTEP-HF trial, which randomly assigned stable symptomatic HFrEF patients (NYHA class II/III) to an equivalent regimen. Sacubitril/valsartan was found to slightly improve the 6MWT by 35.09 m in the OUTSTEP-HF trial, but the treatment difference was not statistically significant [46].

The ACTIVITY-HF trial did not find any treatment differences between sacubitril/valsartan and enalapril, which could be attributed to a multitude of reasons. Individuals in this trial had very low baseline physical activity levels according to mean basal peak VO_2_ values of 12.9 mL/minute/kg for the sacubitril/valsartan group and 13.7 mL/minute/kg for the enalapril group, making them possibly more resistant to intervention than patients with less symptoms [43]. Even in patients with a mean peak VO_2_ of 19 mL/minute/kg, the Brazilian investigation did not find any appreciable variations in peak VO_2_ between sacubitril/valsartan and enalapril [49]. Due to the large number of tests, these results are most likely the product of coincidence; nonetheless, the quantity of daily exercise is strongly connected with exercise capacity and may have affected the change in peak VO_2_ [68,69].

Heavy smokers made up a larger share of the patients in the sacubitril/valsartan group, which may have hampered their ability to exercise more effectively [70]. Improvements in peak VO_2_ and QOL measurements generally showed a favorable connection, according to this comprehensive study. The ACTIVITY-HF trial, in contrast to PARADIGM-HF, demonstrated improvements in KCCQ scores in both treatment arms without a significant difference in the groups. This may be due to changes in the two trials' experimental designs; for example, one of PARADIGM-HF's objectives was the change in KCCQ scores from baseline to eight months [47].

Limitations

This is the first quantitative analysis of sacubitril/valsartan's effects on HRQoL in HFrEF subjects from the standpoint of evidence-based medicine obtained from RCTs only. The Taiwanese researchers factored that heart failure has an irrevocable endpoint and that no therapy may be effective if cardiac fibrosis reaches its final level [59]. As a result, they attempted to administer sacubitril/valsartan to the individuals as quickly as practical [59], albeit they were aware that according to the heart failure treatment recommendations of the 2016 European Society of Cardiology (ESC), sacubitril/valsartan is a comprehensible last resort [71].

The RCTs included in this systematic review and meta-analysis do not exactly pinpoint if the patient had fibrosis set in, as no detailed three-dimensional echocardiography or cardiac magnetic resonance imaging data could be compared, which if available would have fine-tuned the analysis further. In this study, it was not possible to compare whether myocarditis was ruled out as exact data to compare all these RCTs were not adequately available.

The findings, as mentioned in the results above, demonstrate sacubitril/valsartan's positive impact on HRQoL in HFrEF, reinforce the lack of evidence about its influence on the treatment of HFrEF, and add more details for the clinical use of sacubitril/valsartan. However, the meta-analysis performed needs to consider a few possible drawbacks. First, there were some variations in the length of follow-up between studies, which may have had some effect on the comparability. The aggregated effectiveness estimates, however, exhibited promising reliability. Furthermore, there were some restrictions on the consequences that were chosen, which were primarily affected by how the results were reported in research.

Major changes in control medicines, average dose, the number of patients at various dose levels, and NYHA functional class between trials may have resulted in substantial heterogeneity between investigations on some endpoints. It was not possible to perform subgroup analysis due to a lack of sufficient data, even though some studies claimed that several demographic factors, such as age and etiology variations, could have had an influence on the treatment outcome with sacubitril/valsartan.

The potential positive effects of both therapies (the experimental drug and control drugs) on aerobic fitness and its pathophysiological mechanisms should be examined in future trials comprising bigger sample sizes and longer follow-up periods with older adults and more severely impacted individuals with HFrEF.

## Conclusions

This systematic review and meta-analysis study included eight randomized clinical trials. Sacubitril/valsartan did not exponentially improve peak VO_2_ or 6MWT in these trials; however, the patient-reported data suggested that the quality of life was modestly influenced by the drug. The combined effects showed that the sacubitril/valsartan group improved the HRQoL score more than the control group in HFrEF. There were fewer clinical trials to evaluate the minimally significant improvement rate of HRQoL compared to the primary outcome, but the results from individual studies were consistent with the primary outcome's direction, further confirming sacubitril/valsartan's superior HRQoL improvement in HFrEF.

## Appendices

 Table 8 shows the risk of bias.

**Table 8:** Risk of bias Risk of bias coding: low, 1; unclear, 2; and high, 3 EVALUATE-HF, Effect of Sacubitril-Valsartan vs Enalapril on Aortic Stiffness in Patients With Heart Failure and Reduced Ejection Fraction; LIFE-HF, LCZ696 In Hospitalized Advanced Heart Failure; PARADIGM-HF, Prospective Comparison of ARNi With ACE-I to Determine Impact on Global Mortality and Morbidity in Heart Failure; PARALLEL-HF, Prospective comparison of ARNI with ACE inhibitor to determine the noveL beneficiaL trEatment vaLue in Japanese Heart Failure

Name of trial/author	Random sequence generation	Allocation concealment	Blinding of participants	Blinding of outcome assessment	Incomplete outcome data	Selective reporting	Other biases
ACTIVITY-HF trial [43]	1	1	1	1	1	1	2
LIFE-HF trial [50]	1	1	1	1	1	1	2
AWAKE-HF study [44]	1	1	1	1	1	1	2
Dos Santos et. al. [49]	1	1	1	1	1	1	2
OUTSTEP-HF trial [46]	1	1	1	1	1	1	2
PARADIGM-HF [47]	1	1	1	1	1	1	2
EVALUATE-HF [45]	1	1	1	1	1	1	2
PARALLEL-HF [48]	2	2	1	1	1	1	2
